# A research agenda for heritage planning perspectives from Europe, edited by Eva Stegmeijer and Loes Veldpaus. Edward Elgar Publishing Limited, Cheltenham, 2021. 225pp. ISBN 9781788974622

**DOI:** 10.1186/s43238-023-00089-x

**Published:** 2023-04-28

**Authors:** Cut Dewi

**Affiliations:** Department of Architecture and Planning, and International Centre for Aceh and Indian Ocean Studies (ICAIOS), Universitas Syiah Kuala, Jl. Syech Abdulrauf No.7, Darussalam, Banda Aceh 23111 Indonesia


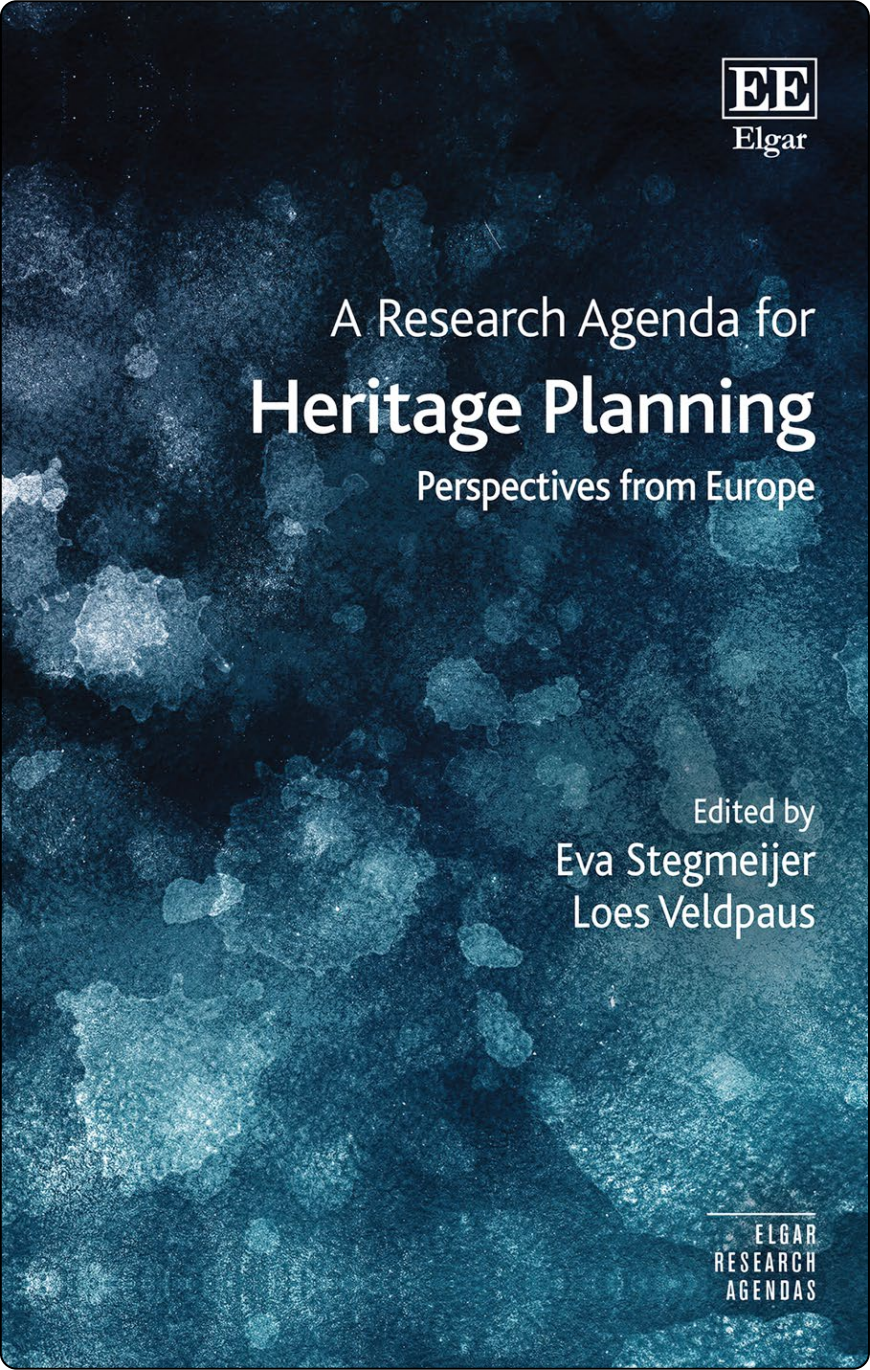
In her book, uses of heritage, Laurajane Smith (2006) mentioned that heritage is a cultural process for amongst others of identity formation within a certain group of people. A closer look at her publication in critical heritage studies suggests that heritage is a work and there is no such thing as heritage without people constantly making, reinterpreting, valorising, and discussing it. The past few decades have witnessed an important critique of the Eurocentric (west) understanding of heritage that potentially masks the heritage process. This critique is mostly, if not all, drawn from a non-western understanding of heritage, especially in Asia (east) (Gao and Jones 2021). Reading *A Research Agenda for Heritage Planning Perspectives from Europe* the book by Eva Stegmeijer & Loes Veldpaus (Stegmeijer and Veldpaus 2021) brings new dialogues and bridges the dichotomy of an ‘east’ and ‘west’ understanding of heritage that has been taken for granted as two different dichotomies. This book offers an insight on how the western world itself is also not homogenous in the understanding of what heritage is and heritage is not always tangible in the ‘west’. This book shows readers that there is no universal European understanding of heritage and planning. Only in specific divisions of European countries and mostly in urban contexts does so-called European heritage understanding dominate the discourse and planning. This book aims to not only elaborate on heritage planning and research in Europe, but also push beyond a Eurocentric approach, and examine the research this approach produces and the foundation on which it is developed, as well as give funding to the projects and people who work in this field.

In addition, readers would also benefit from a more in-depth discussion of new perspectives and interdisciplinary approaches to the heritages present in Europe. This approach, despite different contexts and institutional settings, is also beneficial for readers outside European countries (west). The organisation and topics discussed in the book show Stegmeijer’s and Veldpaus’s observation and interdisciplinary understanding of heritage studies discourses, challenges, and future opportunities. This book discusses a wide range of themes which are of importance to heritage studies, from some general discussions of heritage, identity, and development, to recent trends of discourse heritage as a process, climate change, and the uses of digital media and new technologies in mitigating the effects of climate change on heritage sites and buildings. The book consists of research undertaken in the Netherlands, Norway, Poland, Spain, Serbia, Sweden, and the UK. It highlights three main discourses in heritage studies: heritage and identity, climate, and development; and organises the discussion into three main parts of the book: heritage planning from a European perspective, current research in Europe heritage planning, and research agendas for heritage planning in Europe and beyond.

The first part of the book sets the definition of heritage science as a growing interdisciplinary and cross-cultural discourse. It argues that heritage science is a dynamic sector which draws from diverse humanities, sciences, architecture, tourism, and engineering disciplines and employs the current digital revolution and fosters a deep engagement with society. In addition, this part also shows that the definition of heritage in Europe, especially within the European Union, is getting broader and broader, becoming more inclusive by acknowledging marginalised heritages, and that heritage is not merely a tangible and grand concept. Heritage science has influenced public policies in many areas, including societal, economic and environmental aspects (chapter 1). The results of heritage science can be utilised for the development, implementation, and monitoring of these policies. As many countries have already integrated heritage matters into spatial planning, this has led to heritage issues becoming more important for communities and government alike. There has been a shift over the last couple of decades in city and regional planning from expansion and sprawl to redevelopment and regeneration. The sustainable development discourse has gained momentum in recent years which has led to an increase in the awareness of the uses of environmental sources in which heritage is connected. One important contribution of this book is to introduce and assess culture as a new pillar of sustainable development within an economic, social, and environmental framework (chapter 3). It shows that heritage planning has the potential to generate other economic developments. The book also suggests that to have more inclusive heritage research agenda a move beyond dominant fundings, market frameworks and agendas should be made (chapter 2).

The second part A, current research in heritage planning: Heritage and Identity, highlights the interactions of various people who are identified as ‘normal people’ such as local people with their everyday identity construction, and other heritage stakeholders in participatory heritage practices. This part shows selective uses of ‘local identity’ in regeneration strategies at the regional level and identity politics in which heritage is a part at the European level (chapter 4). In chapter 5, the need to have better communication with local communities in promoting landscape management and conservation in which archaeological sites are part of this integral aspect is argued. Local communities own and use the landscape in their everyday life know the landscape much better than anyone else. However, the authors point out that bottom-up participation and engagement only work in a community where communal decision-making traditions exist. In chapter 6 and 8 of the books, the authors argue that there needs to be a shift in the way we think of heritage for a community from ‘the uses of heritage’ to ‘usefulness of heritage’. Some of the useful aspects of heritage for the EU are to strengthen intercultural dialogue, support economic and social development, and promote social inclusion. This includes the dialogue between local stakeholders and migrants. In chapter 7, the authors suggest participation through digital reconstruction in which local communities get involved in at the beginning of heritage mapping and an exhibition. This involvement will increase their sense of greater belonging to the place, while shedding light on the importance of community participation. At the same time, the book claims that public participation needs a supportive policy at hand.

The second part B discusses the major environmental and social challenges of climate change that are considered in heritage studies. The chapters in this part discuss the uses of technologies in heritage management and mitigating the impact of climate change on heritage (chapter 9). In chapter 10, authors tease out the forgotten roles of waterway heritage, which acts not only as corridors for transportation enabling the spread of human mobility and knowledge as well as trade, but also as spaces of contact, disaster, and geopolitical territorialisation. Thus, water management can minimise the impacts of climate change on cultural heritage, and at the same time, have a positive impact on the connections between cultural and environment, landscape and heritage, and local attitudes and global change. In chapter 11 and 12, the uses of remote sensing technologies in assessing hazards on cultural heritage are discussed. PROTHEGO project (in chapter 11) has applied several remote technologies (radar interferometry) which allows researchers to measure the surface deformation of heritage sites and provide a ranking of the most critical heritage sites across Europe. CLIMA project (chapter 12) not only identifies the threats, but also aims to quantify the possible impact of these threats.

As heritage as a social and cultural process Smith (2006), the second section part C suggests the transformative aspects of heritage-led projects for development and how they can become a node for social and human capital as well as economic development (chapter 13). In spatial planning, heritage sites had to be protected, but now, they are considered assets for development, especially as the globalised economic community grows. For positive outcomes in the development, heritage sites should be used sensitively and sustainably. These examples can be seen in Chapter 14 which describes the use music festivals as fluid heritage, development assets, and as social nodes that give equal places for marginalised groups to take part and develop their own cultural activities. Chapter 15 have suggested possible ways to make a sustainable use of heritage. For them, prevention plans should be developed prior to implementation in order to control the intersection between heritage and environment. This prevention includes the changing attitudes of the owners, policy makers, and other stakeholders from emergency to prevention action and engaging them in integrated projects. In Chapter 16, the authors give examples of how integrated efforts of collaboration between entrepreneurs and policy makers in promoting gastronomy and tourism through taste, culture, and production can boost the economic prospects of heritage.

Finally, as Akagawa (2016) mentions there is no perfect mechanism for heritage conservation. ‘West’ or ‘east’ are just different people and cultural settings, thus resulting in different interpretations. What remains important is that for heritage practitioners, policy makers, communities, or wherever they may be; a dialogue is had with the diverse communities to heritage conservation in mind (Akagawa 2016). In addition, it needs to be understood that an authoritative understanding of heritage, Authorised Heritage Discourse (AHD) (Smith 2006) which is heavily influenced by the ‘west’ has no clear boundaries in the ‘east’ (Dewi et al. 2019). This book presents ongoing dialogues between the ‘east’ and ‘west’ understanding of heritage and proposes alternative and interdisciplinary approaches to heritage studies and encourages future studies in heritage. As global challenges are getting bigger ranging from development, climate change, natural disasters, and the recent Covid-19 pandemic, heritage can play very important roles in societal challenges. It is not a passive victim waiting to be rescued in the face of these challenges.

In the future works in heritage planning and research, in chapter 17, the book suggests not only to ask the ‘how’ of heritage planning, but also to understand what heritage does and to whom, as well as define our expectations of what heritage planning can do. It challenges the participatory approach to not only tick the right boxes, but rather we need to enforce participatory governance through fundamental, valuable, and ethical means by maintaining power relations amongst stakeholders. Within the collaboration between these stakeholders, we should allow more nuanced heritage interpretations and contestations, and embrace dissonance. In the face of mobility, how do we negotiate between place-based heritage and on-the move histories and heritage. Further studies in heritage science will perhaps extend the book’s effort to bring international dialogue on east and west understandings of heritage. Future research might fill the gaps of this book in its limitations in understanding of the destruction of post-disaster heritage sites. In fact, the book does explain how climate change and the ways managing climate change affects cultural heritage, especially the tangible ones using complicated and well-developed technologies. However, a broader understanding of the sudden and tremendous destruction and adaptation of disaster threats and unavoidable impacts of climate change to heritage sites is needed. Besides managing and preventing physical destruction, rethinking alternative solutions for coping with heritage destruction which is unavoidable is also needed. In addition, within this coping mechanism, a much wider and deeper understanding of the impact of these destructions and changes to humanity or society is needed due to the lack of research and understanding. As the book suggests, besides dealing with mitigation and the recovery of heritage sites, societies who are connected with these sites need to have an adaptation scenario, in place should the worst occur. Even to get ‘curated decay’, the destruction and ruination are under consideration in heritage management (DeSilvey 2017) or probably communities may have to implement ‘adaptive reform’ in which destroyed heritage sites might be rebuilt in different forms, but still serve the same functions (Dewi 2017). Accepting these changes, however, the book posts further questions: do we let the world continue without heritage planning, conservation, and a plan for saving these heritage sites? To what extent do we accept the change? Whose heritage will be saved in the face of destruction and other challenges? What kind of policies do communities need to deal with these dynamic changes and what kind of plans should we make to adapt to the worst-case scenarios? Should we also adapt our institutions and regulations to govern these new dynamics? Thus, the book is very potential not only for academics, but also for policy makers, practioners, students, Non-Governmental Organisations (NGO), and community.

## Data Availability

Not applicable.

## Abbreviations

AHD: Authorised Heritage Discourse
PROTHEGO: PROTection of European Cultural HEritage from GeO-hazards
CLIMA: Cultural Landscape risk Identification, Management and Assessment
NGO: Non-Governmental Organisations

## References

[CR1] Akagawa, N. 2016. Rethinking the Global Heritage Discourse – Overcoming ‘East’ and ‘West’? *International Journal of Heritage Studies* 22 (1): 14–25. 10.1080/13527258.2015.1068213.

[CR2] DeSilvey, C. 2017. *Curated Decay Heritage beyond Saving*. Minneapolis: Univ Of Minnesota Press.

[CR3] Dewi, C. 2017. Rethinking architectural heritage conservation in post-disaster context. *International Journal of Heritage Studies* 23: 587–600. 10.1080/13527258.2017.1300927.

[CR4] Dewi, C., Izziah, E. Meutia, and J. Nichols. 2019. Negotiating Authorized Heritage Discourse (AHD) in Banda Aceh after reconstruction. *Journal of Architectural Conservation* 25 (3): 211–227. 10.1080/13556207.2019.1635768.

[CR5] Gao, Q., and S. Jones. 2021. Authenticity and heritage conservation: seeking common complexities beyond the ‘Eastern’ and ‘Western’ dichotomy. *International Journal of Heritage Studies* 27 (1): 90–106. 10.1080/13527258.2020.1793377.

[CR6] Smith, L. 2006. *Uses of Heritage*. New York: Routledge.

[CR7] Stegmeijer, E., and L. Veldpaus. 2021. *A research agenda for heritage planning perspectives from Europe*. Massachusetts: Edward Elgar Publishing Inc.

